# Complex Regional Pain Syndrome with Aortic Distress after Thoracic Endovascular Aortic Repair and False Lumen Exclusion with “Candy Plug” Technique

**DOI:** 10.1055/s-0041-1730007

**Published:** 2021-10-13

**Authors:** Valerio S. Tolva, Andrea Kahlberg, Luca Bertoglio, Santi Trimarchi, Riccardo Miloro, Renato Casana, Roberto Chiesa

**Affiliations:** 1 Department of Vascular Surgery, Grande Ospedale Metropolitano Niguarda, Milan, Italy; 2 Department of Vascular Surgery, Vita-Salute San Raffaele University, Milan, Italy; 3 Department of Vascular Surgery, Fondazione Ca' Granda Ospedale Maggiore Policlinico, Milan, Italy; 4 Department of Clinical and Community Sciences, University of Milan, Milan, Italy; 5 Laboratory of Vascular Research, Istituto Auxologico Italiano, Milan, Italy; 6 Department of Surgery, Istituto Auxologico Italiano, Milan, Italy

**Keywords:** aortic dissection, TEVAR, aortic pain, acute aortic syndrome, candy plug, spinal cord protection, visceral perfusion

## Abstract

A 41-year-old male presented for pain treated with oxycodone. A zone-2 thoracic endovascular aortic repair with distal PETTICOAT (provisional extension to induce complete attachment) for complicated Type-IIIB aortic dissection was performed 18 months before. Repeated hospitalizations did not show any issues to justify the recurrent pain. The aortic nature of the pain was suspected considering the plug as a pain trigger. Through a left thoracoabdominal incision in the eighth intercostal space, the candy plug was removed. Pain diminished after thoracoabdominal surgery steadily.

## Introduction

Aortic dissection is a painful and dangerous condition leading to urgent medical and surgical treatment. False lumen perfusion remains one of the unmet needs after thoracic endovascular aortic repair (TEVAR) and further procedures aim to complete the result. Candy plug technique has been recently introduced as an ancillary operation during TEVAR for Type B aortic dissection. 1 2

Complications are described as the possibility of vessel wall injury caused by continuous shear stress from the plug. 3 Despite the presence of adverse events related to possible mechanical lesions, the review of the literature lacks chronic pain after “candy plug.” 4 5

## Case Presentation



**Video 1** Reporting the surgical approach, candy plug removal, cold renal and visceral perfusion, and result.


**Fig. 1:**
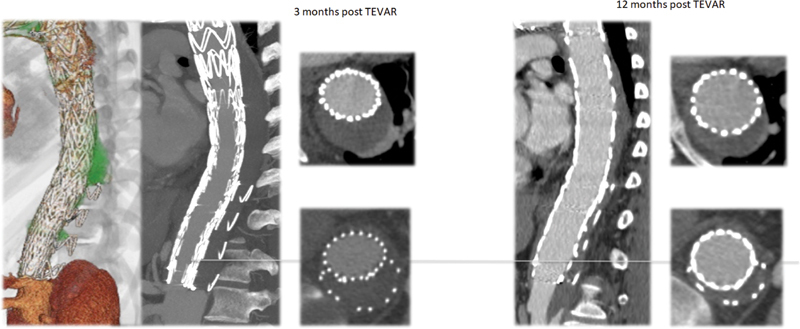
Computed tomography (CT) post- thoracic endovascular aortic repair and “candy plug” CT scan showing effective resolution of Type B aortic dissection.

**Fig. 2:**
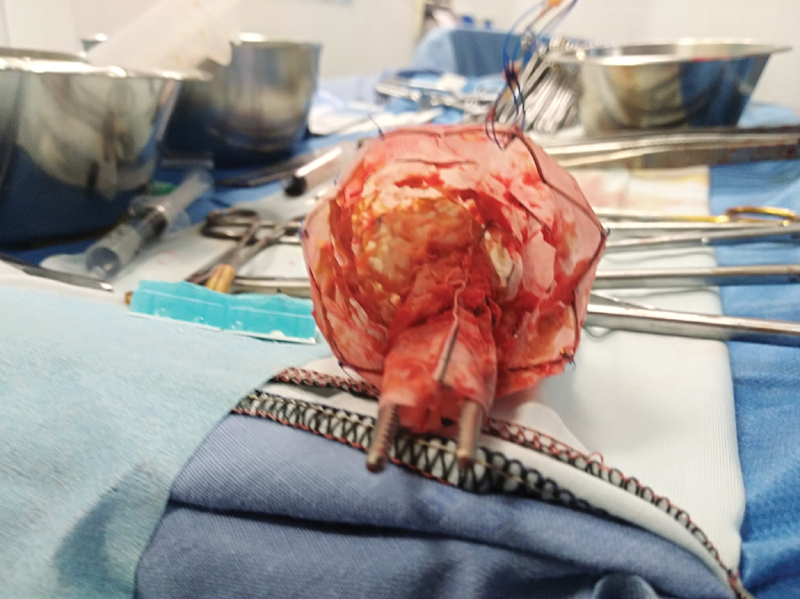
Candy plug after removal. To note, the whole device caliber and the forceps.

Our experience reports the case of a 41-year-old male patient who presented to our outpatient department for severe back pain. The patient had been emergently treated with a zone-2 TEVAR with distal PETTICOAT (provisional extension to induce complete attachment) limited to the thoracic region for complicated Type-IIIB aortic dissection performed due to a worsening of pleural effusion and to a not responding arterial hypertensive state. The left subclavian artery was neither revascularized nor occluded at the origin. The intervention was complicated by left cerebral ischemia; the stroke resulted in a right hemisyndrome with mild walking impairment. Patient followed a neurological rehabilitation program both in hospital and after discharge. A 2-month computed tomography (CT) scan observed a Type-IC endoleak and complete reperfusion of the false lumen from distal reentry tear with enlargement of the false lumen associated with intermittent pain. The subsequent treatment entailed a stent–graft extension from the previous TEVAR to just above the celiac trunk origin followed, 2 weeks after, by embolization of the origin of the left subclavian artery and occlusion of the distal false lumen with a candy plug version II. 2 3 The immediate postoperative course was characterized by a postimplant syndrome treated with steroids. Nonetheless, a few months after these procedures, the patient returned with worsening back pain that was hardly responding to any common pain killers. Repeated hospitalizations and complete vascular and neurological evaluations did not show any technical and anatomical issues to justify the recurrent pain. Follow-up CT scans, performed to rule out an aortic etiology of the pain, revealed a progressive complete thrombosis of the false lumen with progressive shrinkage of its portion in the thoracic region and stable transaortic diameter in the abdominal region with the residual dissection (46 mm; Fig. 1 ). The patient was referred for algological therapy with a mild regression of the visual analogue scale (VAS) from 8 to 6. After 18 months, since the first procedure, the patient was under transdermal oxycodone treatment. A Short Form 12 Health Survey Scale (SF-12) was administered underlying severe physical and social impairment because of back pain and drug therapy. During a new hospitalization, in our hospital, new angio-magnetic resonance imaging (MRI), electroencephalogram, electroneuromyography, and somatosensorial-evoked response tests excluded any neurologic defects. Lung and pleural evaluations showed normal results. In the end, despite the technical success, the aortic nature of the pain was suspected and zooming the bulk of endovascular material inside the thoracic aorta as a trigger for complex regional pain syndrome (CRPS). 4 5 Therefore, the candy plug removal was considered to reduce the radial force working inside the aorta. The patient was approached in two stages. First, a left carotid subclavian artery bypass was performed to increase the collateral network inflow considering that preoperative MRI and CT scan failed to detect patent intercostal arteries arising from the stent–grafted region. Ten days after cervical bypass, a semiconservative open conversion was performed through a left thoracoabdominal incision in the eighth intercostal space; the progressive shrinkage of the thoracic false lumen allowed treating the patient as a Type-IV thoracoabdominal aortic aneurysm (TAAA). Cerebrospinal fluid drainage and permissive hypothermia (lower rectal temperature of 33°C) combined with the previous left subclavian artery revascularization were employed as adjuncts for spinal cord protection. The thoracoabdominal aorta was prepared from approximately 10 cm above the aortic hiatus down to the aortoiliac bifurcation. Before opening the aorta, the region where “candy plug” imprinting the aortic adventitia was evident ( Video 1 ; available in the online version). “Candy plug” bulged out from the aortic false lumen through a 10-cm long longitudinal incision ( Video 1 ; available in the online version). After aortic cross-clamping, the false lumen was longitudinally opened, the candy plug was removed ( Fig. 2 ), and the true lumen endograft was partially resected to both perform the proximal anastomosis in a dissection-healed thoracic region and resect the residual and aneurysmal abdominal aorta. Visceral and renal protection was obtained with local hypothermic perfusion with 4°C Mannitol and Ringer Lactate solution. 2 3 Proximal aortic graft was sutured end to end with the endograft and distally a beveled anastomosis, including visceral and renal vessels, was performed. 3 Recovery was uneventful, and patient was discharged on postoperative day 15. After 4 months, the patient reported VAS 2 with no need of opiates as painkiller.

## Discussion

Chronic pain is an abnormal process due to pain response to the activation of fibers for innocuous stimuli. It is characterized by three processes in the spinal cord accounting for alteration in the somatosensory system, that is, increased excitability, decreased inhibition, and structural reorganization. Their concurrent contribution could lead to CRPS. 4 5 Except for acute aortic syndrome, thoracic aorta is not usually considered as a pain-producing organ. The occurrence of pain during balloon inflation for aortic coarctation or TEVAR, which disappears immediately after deflation, is related to the activation of the orthosympathetic nervous system. When the nervous fibers are triggered, patients experience tachycardia, profuse sweating, and VAS > 8. As soon as the triggering event fades away, the relief for the patient is immediate. Aortic pain is usually associated with dissection and high pressure in the false lumen. Successful exclusion of the false lumen in chronic dissection remains a challenge. Survival is associated with aortic remodeling which is related to the persistence of flow in the false lumen. In literature, a few articles concern candy plug and its application in modern endovascular therapy. 6 The most important search engines related to scientific publications have no more than 30 articles about this topic. We did not find case reports of conversion into open surgery and any complications that arise with the procedure can be treated by conservative therapy or adjunctive endovascular treatment. No pain induced or postimplantation syndromes are related to candy plug. Meanwhile, systemic effects, as a noninfectious fever, elevated C-reactive protein, leukocytosis, and coagulation disturbances are discussed, and ascribed to proinflammatory mediators, description of local effects is missing. 1 One of the considerations about vascular pain is the postoperative modification of sympathetic nervous system with changes in vascular reactivity and morphology leading to CRPS.

Literature has a lack of models of pain after candy-plug insertion, and assumptions are made on basic physiology. Angioplasty procedures could account for pain during the balloon inflation disappearing after deflation. We supposed the delivery of the candy plug associated with the space occupied by the previous endoprosthesis stretched the vessel wall in a chronic pattern. Decision-making was challenging. Patient compliance with pain was very low and functional impairment was practically complete. The aortic stress on the vessel wall was the only option left for neuropathic pain leading us to open treatment. The patient, whom we decided to treat, developed an invalidating CRPS. Open surgery showed how the adventitia layer was under additional stress because of the bulk of the candy plug. As we expected, the pain diminished after the surgery steadily and now the patient is free from painkillers. Complementary lyses of nerve endings during vessel isolation could have shut down the pain loop as it proved in sympathectomy procedures. 7 8
